# Pasireotide is more effective than octreotide, alone or combined with everolimus on human meningioma *in vitro*

**DOI:** 10.18632/oncotarget.19517

**Published:** 2017-07-24

**Authors:** Thomas Graillon, David Romano, Céline Defilles, Christophe Lisbonis, Alexandru Saveanu, Dominique Figarella-Branger, Pierre-Hugues Roche, Stéphane Fuentes, Olivier Chinot, Henry Dufour, Anne Barlier

**Affiliations:** ^1^ Aix Marseille Univ, CNRS CRN2M UMR7286, Marseille, France; ^2^ APHM, La Timone Hospital, Department of Neurosurgery, Marseille, France; ^3^ APHM, La Conception Hospital, Molecular Biology Laboratory, Marseille, France; ^4^ APHM, Nord Hospital, Department of Neurosurgery, Marseille, France; ^5^ APHM, La Timone Hospital, Department of Anatomopathology and Neuropathology, Marseille, France; ^6^ Aix Marseille Univ, INSERM, CRO2 UMR911, Marseille, France; ^7^ APHM, La Timone Hospital, Department of Neuro-oncology, Marseille, France

**Keywords:** meningioma, merlin, mTOR, pasireotide, somatostatin

## Abstract

Pasireotide is a somatostatin analog (SSA) that targets somatostatin receptor subtype 1 (SST1), SST2, SST3, and SST5 with a high affinity. Pasireotide has a better antisecretory effect in acromegaly, Cushing's disease, and neuroendocrine tumors than octreotide. In this study, we compared the effects of pasireotide to those of octreotide *in vitro* on meningioma primary cell cultures, both alone and in combination with the mTOR inhibitor everolimus. Significant mRNA expression levels of SST1, SST2, and SST5 were observed in 40.5%, 100%, and 35% of meningioma samples, respectively. Pasireotide had a significantly stronger inhibitory effect on cell proliferation than octreotide. The effect of pasireotide, but not of octreotide, was significantly stronger in the group expressing the highest level of SST1 mRNA. Combined treatment with pasireotide and everolimus induced a higher reduction in cell viability than that with octreotide plus everolimus. Moreover, pasireotide decreased Akt phosphorylation and reversed everolimus-induced Akt hyperphosphorylation to a higher degree than octreotide. Using 4E-BP1 siRNA (si4E-BP), we demonstrated that 4E-BP1 protein silencing significantly reversed the response to everolimus, both alone and in combination with SSAs. Moreover, si4E-BP completely reversed the inhibition of cyclin D1 expression level and the increase in p27kip1 induced by SSAs, both alone and in combination with everolimus. Our results strongly support the need for further studies on the combination of pasireotide and everolimus in medical therapy for meningiomas.

## INTRODUCTION

The strong expression of somatostatin receptor subtype 2 (SST2) is well described in meningiomas and is used in clinical practice for differential diagnosis involving the use of radiolabeled octreotide SPECT scanning [1–4]. We had previously demonstrated that SST2 expression is found in 100% of meningiomas at a highly variable level [5].

Octreotide is a specific SST2 agonist currently used in somatotroph and neuroendocrine tumors to suppress hormonal hypersecretion and control tumor growth [6–8]. Pasireotide, which is a more recently developed somatostatin analog (SSA), is considered to be a pan-somatostatin agonist due to its high affinity for SST1, SST2, SST3, and SST5 [9–13]. Pasireotide has been demonstrated to have a better antisecretory effect than octreotide in acromegaly, Cushing's disease, and neuroendocrine tumors [13, 14]. The high affinity for SSTs other than SST2 could be responsible for the increased efficacy of pasireotide.

The Pi3K-Akt-mTOR pathway is activated in meningiomas [15]. The mTOR inhibitor everolimus has been shown to inhibit meningioma cell proliferation *in vitro* [16] and meningioma xenograft growth in nude mice [17]. However, mTOR inhibitors in monotherapy have shown limited efficacy in several cancers. This observation could be attributed to feedback activation of the Pi3K pathway, leading to the incomplete inhibition of mTORC_1_-dependent protein synthesis [18, 19]. We have previously demonstrated that octreotide can abrogate the positive feedback induced by rapalogs on human meningiomas and that co-treatment with everolimus and octreotide was more efficacious than treatment with each drug alone [16].

In this study, we compared the characteristics of the pan-SSA pasireotide to those of octreotide *in vitro*, both alone and in combination with everolimus, in a large series of meningioma samples in correlation with somatostatin receptor expression patterns. In addition, we analyzed the transduction pathways and identified 4E-BP1 (eIF4E-binding protein blocking cap-dependent translation) as a key element in the cellular response to combination treatment.

## RESULTS

### SST mRNA expression

**Figure 1:**
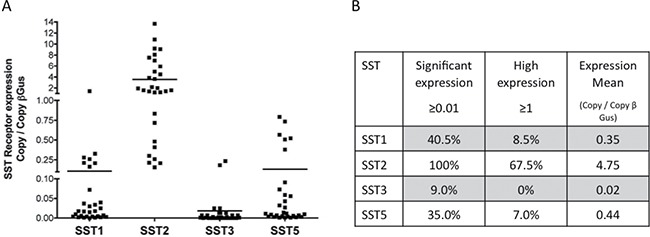
Quantification of SST1, SST2, SST3, and SST5 mRNA expression levels in 30 meningioma samples with variable WHO grades The SST mRNA expression level was measured by real-time PCR and is expressed as copy number/β-Gus. (**A**) Individual values of each tumor. Bars represent the mean. (**B**) SST1, SST2, SST3, and SST5 mRNA expression levels in 30 meningioma samples. Tumors were classified according to the SST mRNA expression level [20]. Percentage of tumors in each category.

The mRNA expression levels of SST1, SST2, SST3, and SST5 were quantified by real-time PCR in 30 meningioma samples, including 16 that were classified as WHO grade I, 11 as WHO grade II, and 3 as WHO grade III (Figure 1, Supplementary Table 1).

All tested meningioma samples expressed a significant (i.e., ≥ 0.01) SST2 mRNA expression level. The mRNA expression level was high (> 1) [21] in 67.5% of the samples. The SST1 mRNA expression level was significant in 40.5% of the samples and high in 8.5% of the samples. The SST5 mRNA expression level was significant in 35% of the samples and high in 7% of samples (Figure 1A, 1B). A significant SST3 mRNA expression level was found in only 9% of the samples. No relationship was observed between SST mRNA expression levels and the WHO grade or Ki67 index.

### Pasireotide induced a higher reduction in cell viability than octreotide

**Figure 2:**
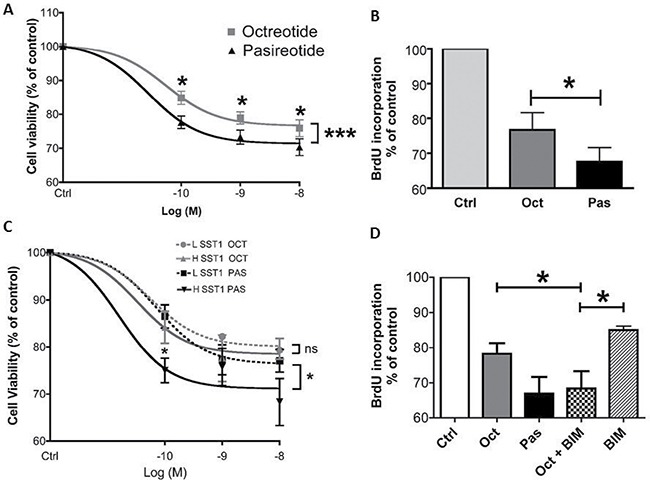
Pasireotide provided a significantly higher dose-dependent reduction in meningioma cell viability than octreotide (*p* < 0.001) The effect of pasireotide was partly dependent on SST1. (**A**) Dose–effect curve of octreotide *vs*. pasireotide (10^−10^ to 10^−8^ M) upon treatment for 3 days on cell viability estimated using a cell viability assay (Cell Titer Glo) in 27 octreotide- and pasireotide-responsive meningioma samples. Results are expressed as mean ± SEM percentage of cell viability *vs*. controls; *p < 0.05, ***p < 0.001. (**B**) Effect of octreotide and pasireotide treatment at 10^−9^ M for 2 days on cell proliferation analyzed by BrdU incorporation in 10 octreotide- and pasireotide-responsive meningioma samples with variable WHO grades (5 WHO grade I, 4 WHO grade II, and 1 WHO grade III). Results are expressed as mean ± SEM percentage of BrdU incorporation *vs*. controls; *p < 0.05. (**C**) Nineteen meningioma samples were separated into two groups depending on the SST1 mRNA expression level [group LSST1 (*n* = 8) with low SST1 mRNA expression (< 0.1 copy/β-Gus) and group HSST1 (*n* = 11) with high SST1 mRNA expression (≥ 0.1 copy/β-Gus)]. Dose–effect curves of the LSST1 group *vs*. the HSST1 group in the presence of octreotide *vs*. pasireotide (from 10^−10^ to 10^−8^ M) on cell viability estimated by Cell Titer Glo. Results are expressed as mean ± SEM percentage of cell viability *vs*. controls; *p < 0.05, ns: not significant. (**D**) Although octreotide was less effective, combined treatment with the SST1 agonist (BIM23926, 10^−7^ M) and octreotide had the same inhibitory effect as treatment with pasireotide (10^−9^ M) on BrdU incorporation for 2 days in three meningioma samples; *p < 0.05. oct: octreotide, pas: pasireotide, ever: everolimus, BIM: BIM23926.

The inhibitory effects of increasing doses of pasireotide were compared to those of octreotide in 34 randomly selected meningioma samples, including 22 WHO grade I, 10 WHO grade II, and 2 WHO grade III samples of various subtypes (Figure 2A, Supplementary Table 1). Pasireotide and octreotide reduced cell viability in the primary cells cultures of 32/34 (94%) and 28/34 (81%) samples, respectively. Only one sample that was responsive to octreotide did not respond to pasireotide. The maximum reduction in cell viability observed at 1 or 10 nM was variable without any relationship with the WHO grade or Ki67 index. In 27 octreotide- and pasireotide-responsive tumor samples, the mean inhibition for each tested dose (from 0.1 to 10 nM) was significantly better for pasireotide than for octreotide [(e.g., at 1 nM, −26% ± 0.5% for pasireotide and −22% ± 0.5% for octreotide (*p* < 0.0003; Figure 2A)]. Pasireotide induced significantly better inhibition than octreotide in 22/27 tumor samples, whereas octreotide induced a significantly better inhibition than pasireotide in 5/27 tumor samples (Supplementary Table 1).

The improved efficacy of pasireotide compared to that of octreotide was confirmed by BrdU incorporation into 11 randomly selected tumor samples with various WHO grades (Figure 2B, Supplementary Table 1). Pasireotide reduced BrdU incorporation in all tumor samples, whereas octreotide reduced BrdU incorporation in only 10 of them. In the 10 octreotide- and pasireotide-responsive tumor samples, the mean BrdU incorporation inhibition at 1 nM was −36% ± 3% for pasireotide and −26% ± 3% for octreotide (*p* < 0.05), highlighting that pasireotide had a stronger antiproliferative effect than octreotide (Figure 2B).

Pasireotide, octreotide, or everolimus, alone or in combination, did not induce apoptosis in the tested tumor samples as demonstrated by terminal deoxynucleotidyl transferase-mediated nick end labeling (TUNEL) assay or by the determination of caspase-3 and -7 activities (Supplementary Figure 1; for tumor characteristics, see Supplementary Table 2).

### Pasireotide inhibition was partially dependent on SST1 mRNA expression level

Considering the high affinity of pasireotide for SST1 and SST5, the impact of the mRNA expression level of these receptors on pasireotide responsiveness was compared to that on octreotide responsiveness. Meningioma samples were classified into the following two groups according to their SST1 or SST5 mRNA expression level: (1) low SST1 or low SST5 mRNA expression (namely, LSST1 or LSST5 group, respectively, < 0.1 copy/β-Gus copy) and (2) high SST1 or high SST5 mRNA expression (namely, HSST1 or HSST5 group, respectively, ≥ 0.1 copy/β-Gus copy). The inhibitory effect of pasireotide was significantly stronger in the HSST1 group than in the LSST1 group (*p* = 0.04), whereas the inhibitory effect of octreotide was not statistically different in both groups (*p* = 0.2, Figure 2C). In contrast, when the tumor samples were classified according to their SST5 mRNA expression levels, the efficiency of pasireotide was not statistically different between the HSST5 and LSST5 groups (*p* = 0.68, Supplementary Figure 2). The SST2 mRNA expression level was not significantly different between the HSST1 and LSST1 groups (*p* = 0.07) and between the HSST5 and LSST5 groups (*p* = 0.7). To confirm the involvement of SST1 in pasireotide responsiveness, the response to pasireotide was compared to that of the combination of octreotide plus an SST1 agonist (BIM23926). The SST1 agonist induced a slight decrease of 15% in cell viability. The combination of octreotide and the SST1 agonist had a similar inhibitory effect on BrdU incorporation as pasireotide alone, whereas octreotide-induced inhibition was significantly weaker (−22% ± 3% for octreotide, −33% ± 5% for pasireotide, and −32% ± 5% for octreotide plus BIM23926; *p* < 0.01; Figure 2D). Together, these data suggest that SST1, but not SST5, is involved in the increased efficacy of pasireotide on BrdU incorporation in comparison to octreotide in meningioma samples.

### Combined treatment with pasireotide and everolimus induced a higher reduction in cell viability than that with octreotide and everolimus

**Figure 3:**
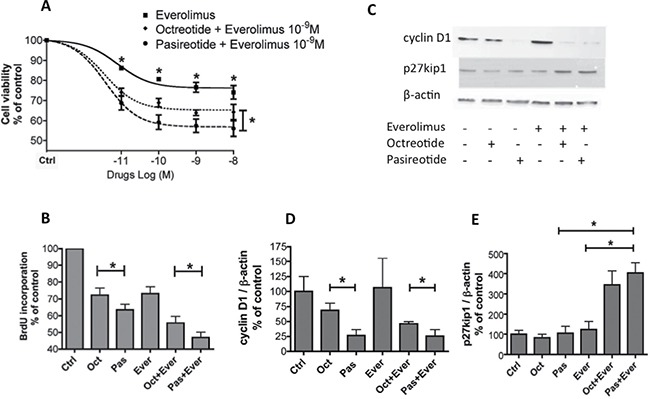
Combination treatment of pasireotide with everolimus resulted in a significantly higher dose-dependent reduction in meningioma cell viability than combination treatment with octreotide and everolimus (**A**) Dose–effect curve of everolimus alone or in combination with octreotide or pasireotide (10^−10^ to 10^−8^ M) upon treatment for 3 days on cell viability estimated using the Cell Titer-Glo assay from seven meningioma samples including four WHO grade I and three WHO grade II samples. Results are expressed as mean ± SEM percentage of cell viability *vs*. controls (nontreated cells); *p < 0.05. (**B**) Effect of everolimus, octreotide, or pasireotide at 10^−9^ M, alone and with combined treatment for 2 days, on BrdU incorporation in eight meningioma samples with variable WHO grades (four WHO grade I, three WHO grade II, and one WHO grade III). Results are expressed as mean ± SEM percentage of BrdU incorporation *vs*. controls; *p < 0.05. (**C**, **D**, **E**) Western blot analysis of cyclin D1, p27kip1, in eight meningiomas (six WHO grade I and two WHO grade II) after 3-h incubation with 10^−9^ M everolimus, octreotide, or pasireotide alone, or with combined treatment. (C) Immunoblot of a representative tumor M49. (D, E) Quantification of cyclin D1/β-actin (D) and p27kip1/β-actin (E) from the immunoblot. The results are represented as the mean percentage of control (nontreated cells) obtained from the eight tumors; *p < 0.05. oct: octreotide, pas: pasireotide, ever: everolimus, ctrl: control.

Next, we compared the effect of the combined treatment with pasireotide and everolimus to that with octreotide and everolimus on cell viability in seven meningioma samples, including four WHO grade I and three WHO grade II samples. First, as we have previously shown for octreotide, combined treatment with pasireotide and everolimus caused a significantly higher reduction in cell viability than that by everolimus alone at each tested dose (Figure 3A, Supplementary Table 1). Moreover, combined treatment with pasireotide and everolimus caused a significantly higher reduction in cell viability than that with octreotide and everolimus (*p* < 0.05, Figure 3A, Supplementary Table 1). The mean maximum reduction in cell viability was −25% ± 3% for everolimus alone, −36% ± 2% for everolimus and octreotide, and −45% ± 2 % for everolimus and pasireotide (Figure 3A and Supplementary Table 1).

The results using BrdU incorporation on eight tumor samples (four WHO grade I, three WHO grade II, and one WHO grade III) confirmed that combined treatment with pasireotide and everolimus was more efficacious than treatment with everolimus alone and in combination with octreotide and everolimus (*p* < 0.05, Figure 3B, Supplementary Table 1). The mean reduction in cell proliferation was −27% ± 4% for everolimus, −46% ± 3% for combined treatment with everolimus and octreotide, and −54% ± 2% for combined treatment with everolimus and pasireotide (*p* = 0.001, Figure 3B and Supplementary Table 1).

We next assessed the expression levels of cyclin D1 and p27kip1, which are two proteins involved in the cell cycle, that are modulated by SSAs in neuroendocrine tumors [18] and expressed in meningiomas [21, 22] by western blotting in eight meningioma samples (six WHO grade I and two WHO grade II). Alone or in combination with everolimus, pasireotide decreased cyclin D1 expression to a higher degree than octreotide (*p* < 0.05, Figure 3C, 3D). Combined treatment with everolimus and pasireotide and with everolimus and octreotide strongly increased p27kip1 expression levels (*p* < 0.05, Figure 3C, 3E).

### Effect of pasireotide and octreotide, alone or in combination with everolimus, on the Pi3K-AkT-mTOR and ERK pathways

**Figure 4:**
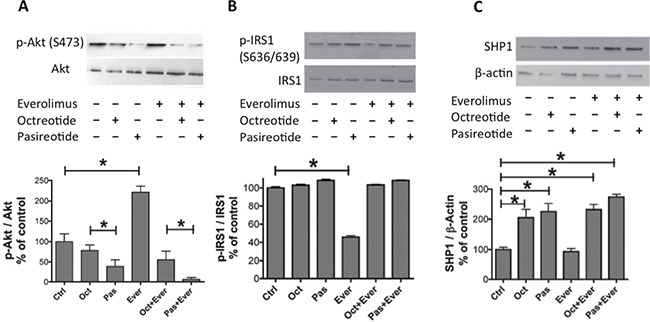
Pasireotide and octreotide reversed the everolimus-induced increase in the Akt phosphorylation level, decrease in the IRS1 phosphorylation level, and increase in the SHP1 expression level Western blot analysis of phospho-Akt on Ser^473^ (p-Akt), total Akt (**A**), phospho-IRS1 (p-IRS1) and total IRS1 (**B**), and SHP-1 and β-actin (**C**) after 3-h incubation with 10^−9^ M pasireotide, octreotide, or everolimus, alone or in combination. The representative immunoblots of quantifications in A, B, and C are those of the M53 tumor. (A) Quantification of p-Akt/Akt from the immunoblot. The results are represented as the mean percentage of controls (nontreated cells) obtained from eight tumors (six WHO grade I and two WHO grade II). (B) Quantification of p-IRS1/IRS1 from the immunoblot. The results are represented as the mean percentage of controls obtained from three tumors (one WHO grade I and two WHO grade II). (C) Quantification of SHP1 and β-actin from the immunoblot after immunoprecipitation. The results are represented as the mean percentage of controls obtained from four tumors (one WHO grade I and three WHO grade II); *p < 0.05. oct: octreotide, pas: pasireotide, ever: everolimus.

First, we characterized the effect of the activation of positive feedback on the Akt/Erk oncogenic pathway with everolimus treatment in the meningioma samples. Everolimus induced an increase in the phosphorylation level of Akt (*p* < 0.05, Figure 4A) and a decrease in the phosphorylation level of IRS1 (*p* < 0.05, Figure 4B) but had no impact on the phosphorylation levels of ERK1/2 (Supplementary Figure 3). In contrast, pasireotide induced a decrease in Akt phosphorylation levels and reversed the everolimus-induced hyperphosphorylation of Akt to a higher degree than that observed for octreotide (*p* < 0.05, Figure 4A). Moreover, octreotide and pasireotide reversed the everolimus-induced decrease in the IRS1 phosphorylation level on Ser^636/639^ (*p* < 0.05, Figure 4B). Octreotide and pasireotide, alone or in combination with everolimus, increased the phosphorylation level of SHP1 (*p* < 0.05, Figure 4C) but had no impact on the phosphorylation levels of ERK1/2 (Supplementary Figure 3).

### 4E-BP1 a crucial factor in the response to combined treatment with everolimus and SSAs

**Figure 5:**
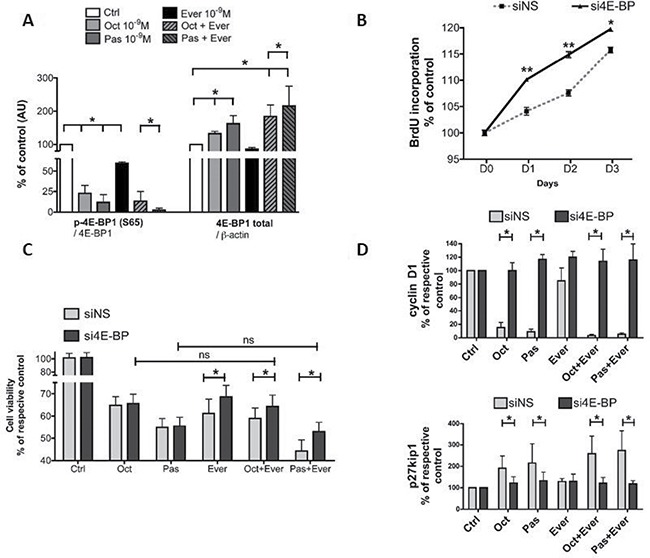
Involvement of 4E-BP1 in response to pasireotide, octreotide, or everolimus alone or in combination (**A**) Western blot analysis of 4E-BP1 phosphorylated on Ser^65^ [p-4E-BP1(S65)] and total 4E-BP1 (4E-BP1) after overnight incubation with 10^−9^ M pasireotide, octreotide, and everolimus, or combined treatment. Quantification of p-4E-BP1(S65) (reported to total 4E-BP1) and total 4E-BP1 (reported to β-actin). The results are represented as the mean percentage of controls (nontreated) obtained from three tumors (two WHO grade I and one WHO grade III); *p < 0.05. oct: octreotide, pas: pasireotide, ever: everolimus. (**B**) Impact of 4E-BP1 siRNAs on the cell proliferation of six meningioma samples (three WHO grade I and three WHO grade II) transfected with either nonspecific (siNS) or 4E-BP1 (si4E-BP) siRNA at 10 nM. Cell proliferation curves for 3 days of siNS cells and si4E-BP1 cells were obtained using BrDU incorporation. The results are represented as the mean percentage at day zero obtained from two tumors; *p < 0.05; **p < 0.01. (**C**) 4E-BP1 blocking by siRNA partially reversed the impact of treatment with 10^−9^ M pasireotide or octreotide combined with everolimus. Cell viability was measured by Cell Titer Glo 48 h after transfection. The results are represented as the mean percentage of each respective control (without drug treatment, ctrl) *p < 0.05. (**D**) 4E-BP siRNA reversed the impact of SSA alone or in combination with everolimus on cyclin D1 and p27kip1 expression levels. Cyclin D1/ β-actin and p27kip1/ β-actin expression levels were analyzed by western blotting. The results are represented as the mean percentage of each respective control. ctrl: control; oct: octreotide; pas: pasireotide; ever: everolimus; *p < 0.05.

Similar to octreotide, pasireotide decreased the 4E-BP1 phosphorylation levels of Ser^65^ (*p* < 0.05, Figure 5A, Supplementary Figure 4A) and Thr^70^ (*p* < 0.05, Supplementary Figure 5). When combined with everolimus, a stronger decrease in the phosphorylation level was observed for pasireotide than for octreotide (*p* < 0.05). Octreotide and pasireotide, alone or in combination with everolimus, increased the 4E-BP1 protein level, although this increase was stronger for pasireotide (*p* < 0.05, Figure 5A, Supplementary Figure 4A).

The increase in the 4E-BP1 protein level associated with the inhibition of 4E-BP1 phosphorylation strongly suggests that 4E-BP1 plays a key role in the response to combined treatment with everolimus and SSAs. To explore this hypothesis, 4E-BP1 was blocked by an siRNA approach in four randomly selected meningioma samples (three WHO grade I and one WHO grade II). The proportion of transfected cells after treatment with fluorescence non-specific siRNA (siNS) was approximately 90% of meningioma cells (Supplementary Figure 6). First, an siRNA complementary to the 4E-BP1 3′-UTR [23] (si4E-BP) was validated for its ability to silence 4E-BP1 expression. Stronger 4E-BP1 inhibition was observed at 48 h following transfection (−65% ± 3%) compared to that with siNS (Supplementary Figure 7A) and was associated with an increase in the protein levels of cyclin D1 (+231% ± 167%) and p27kip1 (+ 402% ± 52%; Supplementary Figure 7B, 7C). BrdU incorporation results showed that cell proliferation over 3 days was significantly higher in si4E-BP cells than in siNS cells (*p* = 0.002 at D1 and D2; *p* = 0.04 at D3, Figure 5B) in two WHO grade II tumors.

4E-BP1 protein silencing had no impact on the response to octreotide or pasireotide but partially reversed the response to everolimus in combination with SSA on cell viability (Figure 5C) and cell proliferation (Supplementary Figure 8). The impact of si4E-BP silencing on the effect of everolimus was not clear. A significant increase was observed using cell viability (Figure 5C) but not BrdU incorporation (Supplementary Figure 8). In fact, with 4E-BP1 protein silencing, the response observed to the combined treatment was similar to that observed with treatment with SSA alone without 4E-BP1 silencing (Figure 5C). Moreover, si4E-BP1 completely reversed the inhibition of cyclin D1 expression and the increase in p27kip1 level induced by SSAs, both alone and in combination with everolimus (Figure 5D, Supplementary Figure 4).

## DISCUSSION

Aggressive and resistant meningiomas remain an unmet medical need in the field of neurooncology, despite the presence of several clinical trials that have tested numerous drugs [24–26]. Octreotide may be a relevant therapeutic agent; however, it is effective primarily in WHO grade I tumors, particularly those at the base of the skull [5]. One clinical study recently tested pasireotide as a therapeutic agent for meningiomas. The efficacy was modest in aggressive meningiomas (recurrent WHO grade II and III meningiomas, PFS6 = 12%) [27]. Recent advances in understanding the physiopathology of meningiomas have identified new therapeutic targets in the Pi3K-Akt-mTOR pathway. We recently demonstrated an *in vitro* additive effect of octreotide and the mTOR inhibitor everolimus on meningiomas [16], leading to an ongoing clinical trial (NCT02333565) [28].

Octreotide is a specific SST2 receptor agonist, whereas pasireotide is a pan-somatostatinergic agonist [9]. We demonstrated for the first time that pasireotide induces a higher reduction in cell viability and stronger inhibitory effect on cell proliferation than octreotide in meningiomas in primary cell cultures. The stronger reduction in Akt phosphorylation and cyclin D1 expression levels provided additional evidence on the improved efficacy of pasireotide. The better antiproliferative activity of pasireotide has been previously established *in vitro* only in human umbilical vein endothelial cells (HUVECs) [29]. Such a better antiproliferative effect has yet to be demonstrated *in vitro* in pituitary and neuroendocrine tumors, which are the usual targets of these drugs [30–32]; however, the better antisecretory activity is clear *in vitro* and *in vivo*. Furthermore, pasireotide has an established antitumoral efficacy *in vivo* in progressive neuroendocrine tumors resistant to octreotide [33].

Pasireotide binds avidly not only to SST2 but also to SST1, SST3, and SST5. The affinities of pasireotide are 30-, 5-, and 40-fold higher than that of octreotide for SST1, SST3 and SST5 respectively [9]. SST1 and SST5 were expressed in 40.5% and 35% of our meningioma samples, respectively, whereas significant SST3 mRNA expression was found in < 10% of samples. In a study by Arena et al., using RT-PCR, SST1 mRNA expression was found in 69% and SST5 mRNA expression in 33% of meningioma samples [34]. In our study, the efficacy of pasireotide appeared to be partly dependent on SST1, whereas no relationship was observed with SST5. In contrast, in corticotroph adenomas and in mammary glands, SST5 and SST3, respectively, could be involved in the better efficacy of pasireotide [35, 36]. SST1 is expressed by fibroblasts, and recently, its role has been implicated in the effect of pasireotide in the microenvironment of pancreatic adenocarcinomas. This effect is mediated by the inhibition of IL6 secretion [37]. Meningiomas are believed to develop from meningiothelial cells that have several functions, including participating in immune response and IL6 secretion [38]. We hypothesize that IL6 secretion from meningioma cells could be involved in the tumorigenesis of meningiomas. Therefore, the antitumoral effect of pasireotide can be amplified by inhibiting this IL6 autocrine/paracrine loop. However, this remains to be explored in future studies.

SST is certainly not the only parameter involved in the better efficacy of pasireotide than octreotide. The better stability of pasireotide [9], the agonist-specific SST2-dependent pathway [39], and agonist-specific SST2 trafficking [40, 41] should also be involved.

Several recent studies have highlighted the potential therapeutic utility of rapalogs in the tumoral control of meningiomas in humans [16, 17]. However, rapalogs used in monotherapy have had only modest success in several cancers. This is probably due to the positive feedback on the Akt oncogenic pathway as described *in vitro* and *in vivo* for different cell types that involves (i) the suppression of inhibitory feedback mediated by the S6K-IRS1-Pi3K loop and (ii) the failure to stop the mTORC_2_-mediated phosphorylation and activation of Akt [15, 42]. In human meningioma cells, we observed an increase in Akt phosphorylation levels and a decrease in IRS1 phosphorylation levels with everolimus, underlining the potential utility of combination therapeutic strategies to block this positive feedback on the Akt oncogenic pathway. We had previously demonstrated that octreotide reversed this positive feedback effect and improved the antiproliferative effects of everolimus on human meningiomas *in vitro* [16]. In the present study, we showed that pasireotide combined with everolimus was more efficient than octreotide combined with everolimus to reduce cell viability, inhibit proliferation and cyclin D1 expression, and reverse the Akt phosphorylation level induced by everolimus. Moreover, a strong increase in the p27kip1 expression level was observed after 3 h of combined treatment, whereas no modification was observed with each drug treatment alone. Akt sequesters p27kip1 in the cytosol by inducing its phosphorylation. The cytosol p27kip1 sequestration favors its degradation. A strong decrease in Akt activity was observed with treatment with SSAs. We hypothesized that this decrease enabled the effect of everolimus on p27kip1, inducing a strong increase in p27kip1 nuclear import [43]. This remains to be elucidated in meningiomas.

Although numerous intracellular pathways have been recognized to mediate the antiproliferative effects of SST, there is a consensus that these effects are mediated by phosphotyrosine phosphatases (PTPs) [44]. SHP1 represents the classical PTP involved in the antiproliferative activity of SSAs [45]. SHP1 activation by SST induces the arrest of cell proliferation in numerous tumor cell lines [45]. In the present study, octreotide and pasireotide increased SHP1 mRNA expression levels (reflecting an increase in SHP1 activity) in meningioma cells. Moreover, the SSAs reversed the everolimus-induced decrease in IRS1 phosphorylation levels on Ser^636/639^, the site primarily phosphorylated by p70S6K. These results are in agreement with those of Cerovac et al. in pituitary tumor cells, wherein they demonstrated that octreotide increased the IRS1 phosphorylation level suppressed by rapamycin through SHP1 [18].

Downstream of the Pi3K-Akt-mTOR pathway, 4E-BP1 is a key factor that mediates the inhibitory effects of rapalogs that induce the inhibition of translation. 4E-BP1 is a binding protein that interacts with eiF4. mTOR activation leads to 4E-BP1 phosphorylation, inducing eiF4-4E-BP1 complex dissociation, eiF4 liberation, and cap protein translation, which results in cell proliferation. Similar to octreotide, pasireotide induced 4E-BP1 hypophosphorylation on Ser^65^ to a higher degree than everolimus. Moreover, both pasireotide and octreotide increased the levels of the 4E-BP1 protein, enhancing the blockage of cap-dependent translation. The double effects of SSA on 4E-BP1 (through the inhibition of 4E-BP1 phosphorylation and increase in 4E-BP1 protein level) together with the inhibition of 4E-BP1 phosphorylation by everolimus suggest a key role of 4E-BP1 in the cooperative effect of SSA plus everolimus on cell proliferation and on cyclin D1 and p27kip1 protein levels. Our experiments using 4E-BP1 silencing by siRNA provided support for this hypothesis, i.e., si4E-BP reversed the additive effect of the combined treatment. The inhibition of cell viability induced by combined treatment with SSA and everolimus after si4E-BP silencing was similar to that induced by SSA alone. Moreover, 4E-BP1 silencing by siRNA completely reversed the drug effects on cyclin D1 and p27kip1 but only partially reversed those on cell proliferation, suggesting an additional transduction pathway for controlling cell proliferation.

We clearly demonstrated that pasireotide has a higher efficacy than octreotide in reducing cell viability and incorporating BrdU in human meningioma cells, both alone and in combination with everolimus. *In vivo*, pasireotide treatment may also have an antitumoral effect through its impact on the tumoral microenvironment, for instance, through VEGF inhibition [46, 47]. VEGF has been demonstrated to be involved in cell proliferation and peritumoral edema of meningiomas [48, 49]. The antiangiogenic effect of pasireotide was higher than that of octreotide in HUVECs *in vitro* [29] supporting the hypothesis of a better efficacy of pasireotide in comparison with octreotide *in vivo*. However, VEGF inhibition by SSAs has to be demonstrated in meningiomas.

Overall, these data highlight the potential utility of the pan-somatostatin molecule pasireotide in treating meningiomas. A clinical trial testing pasireotide alone in WHO grade I meningiomas has reported 50% PFS6, suggesting its potential therapeutic use for treating these tumors [27]. This *in vitro* study on pasireotide in combination with everolimus provides further evidence on the utility of pasireotide in comparison with octreotide, supporting the need for future clinical trials combining pasireotide and everolimus, particularly for treating aggressive tumors.

## MATERIALS AND METHODS

### Materials

Octreotide, pasireotide, and everolimus were obtained from Novartis International AG (Basel, Switzerland). The SST1 agonist (BIM 23926, IC_50_: 12 nmol/l for SST1) was obtained from IPSEN Biomeasure (Boston, MA, USA).

### Primary cell culture of fresh human meningiomas

This study was conducted using human meningioma samples collected from 70 patients (Supplementary Tables 1 and 2). Tumor grading was done according to the WHO 2007 criteria as follows: grade I (*n* = 40), grade II (*n* = 26), and grade III (*n* = 4). The present study was approved by the ethics committee of Aix-Marseille University and was conducted after obtaining informed consent from each patient. Briefly, fresh tumor fragments were minced into pieces smaller than 1 mm^3^ and disaggregated into single cells by exposure to 0.37% type I collagenase (Invitrogen, Cergy-Pontoise, France) for 2 h. The cells were resuspended in complete medium [half DMEM high glucose (4.5 g/l), half F12 media, supplemented with 10% fetal bovine serum (FBV), penicillin (100 U/ml), streptomycin (100 U/ml), and glutamine (100 U/ml)] [50]. The experiments were performed in the first 2 weeks after surgery and before the third subculture. During this time, the tumor cells in the primary culture maintained their SST2 mRNA expression level and response to octreotide [16]. The experiments were performed according to the quantity of tumor cells available after tumor dissociation (Supplementary Tables 1 and 2). SST2 being expressed only by meningioma cells but not by fibroblasts, the lack of fibroblast in the meningioma primary culture was followed by SST2 labeling [5].

### Cell viability

Cell viability was determined by performing luminescent cell viability assay (Cell Titer Glo, Promega Corporation, Charbonnier, France) in triplicate wells containing 2 × 10^4^ meningioma cells. Twenty four hours after plating, the cells were incubated in low-serum media (5% FBV) and treated with drugs (octreotide and pasireotide alone or in combination with everolimus from 10^−10^ to 10^−8^ M) for 3 days. The results are expressed as the mean percentage of cell viability in treated and in nontreated cells. The effect was considered significant when the percentage of cell viability reduction was > 10%.

### BrdU (*5-bromo-2′-deoxyuridine*) incorporation

A total of 4,000 cells were plated in a 96-well plate. After 24 h, the cells were incubated in low-serum media and treated with drugs for 2 days. On the third day, BrdU was added at a final concentration of 1 μM. After incubation for 16 h, DNA synthesis was assayed using a Cell Proliferation ELISA BrdU kit (Roche Molecular, Biochemical, Meylan, France). Newly synthesized BrdU-DNA was determined using a microplate reader (Berthold Technologies, Thoiry, Yvelines, France).

### Apoptosis study

Apoptosis was evaluated by the TUNEL assay using ApopTag Red *In Situ* Apoptosis Detection Kit (Chemicon International Inc, Temecula, CA) and by determining caspase-3 and -7 activities using luminescent Caspase Glo assay (Caspase Glo, Promega Corporation, Charbonnier, France) as previously described [5]. H_2_O_2_ at 50 mM treatment inducing apoptosis was used as a positive control in the caspase experiment and staurosporine treatment (Sigma-Aldrich, Saint Quentin Fallavier, France) at 10^−10^ M in the TUNEL assay and caspase experiments.

### Western blotting and immunoprecipitation

After 24 h, the cells were incubated in low-serum medium for 16 h and then treated with drugs for 3 h, except for 4E-BP1 and its phosphorylated form, which was analyzed after a 16-h incubation period. Meningioma lysates were obtained by mechanical homogenization in a lysis buffer [51]. Total denatured proteins (25 μg) were separated using 10% or 15% SDS-PAGE and were transferred to a polyvinyl difluoride membrane (PerkinElmer, Courtaboeuf, France). After blocking, the membrane was treated with a primary antibody overnight at 4°C, followed by treatment with horseradish peroxidase (HRP)-conjugated secondary antibody. Detection was achieved with the Lumina Forte Western HRP substrate (Millipore, Saint-Quentin-en-Yvelines, France) in GBox (Ozyme, Saint-Quentin-en-Yvelines France).

The primary antibodies were mouse monoclonal antibodies against phospho-p44/42 MAPK (p-ERK), p44/42 MAPK (ERK), phospho-Ser473-Akt [p-Akt(S473)], Akt, 4E-BP1, cyclin D1, p27/Kip1, SHP1(C14H6), IRS1, and phospho-Ser636-Ser639-IRS1 (P-IRS1 S636/639), β-actin, and the rabbit polyclonal phosphospecific antibody p-4E-BP1 directed against Thr 70 [p-4E-BP1 (T70)] or against Ser 65 [p-4E-BP1 (S65)]. All antibodies were purchased from Cell Signalling (Saint-Quentin-en-Yvelines, France). SHP1 proteins were immunoprecipitated using a protein G immunoprecipitation kit (Sigma-Aldrich, France). Immunoprecipitated proteins were analyzed by western blotting with SHP1 antibodies.

### 4E-BP1 siRNA inactivation

To stop 4E-BP1 gene expression, the cells were plated in 6-well dishes, allowed to grow for 24 h, and transfected with 10 nM siRNA targeting 4E-BP1 (Life Technologies, ref. AM16708, France) using an SiPOrt NeoFx transfection reagent (Life Technologies), according to the manufacturer's instructions. Control cells were transfected with silencer Cy-3 negative control siRNA (Life Technologies, Supplementary Figure 6). Gene expression was assessed by western blotting, and the maximum extinguishing of expression was observed at 48 h after transfection (Supplementary Figure 7A). For pharmacological studies, drugs were added 3 h after siRNA transfection.

### Somatostatin receptor mRNAs

The mRNA expression levels of SST1, SST2, SST3, and SST5 were assessed using real-time PCR. Total RNA was extracted from 2.5 × 10^5^ cells and was reverse transcribed into cDNA using Superscript II Reverse Transcriptase (Invitrogen, Cergy-Pontoise, France). The 5′-exonuclease (Taq man) assay was used to quantify SST mRNA, and mRNA expression levels were normalized to β-glucuronidase (β-Gus) mRNA expression levels [52].

### Statistical analysis

Results are presented as mean ± SEM. When the number of samples was less than 30, a nonparametric test was used. The statistical significance between two unpaired groups was determined by the Mann–Whitney nonparametric test, and the statistical significance between two paired groups was assessed by the Wilcoxon nonparametric test. To measure the strength of the association between pairs of variables without specifying the dependency, Spearman rank-order correlations were run. When the number of samples was more than 30, a parametric test (Student's *t*-test) was used. Differences were considered significant at *p* < 0.05.

## SUPPLEMENTARY MATERIALS FIGURES AND TABLES


