# Supplementary material for: Pasireotide is more effective than octreotide, alone or combined with everolimus on human meningioma in vitro
Source: Oncotarget. 2017 Jul 24;8(33):55361–73. doi: 10.18632/oncotarget.19517 (PMC5589664; doi:10.18632/oncotarget.19517)
Supplement: Supplementary file 1 [file oncotarget-08-55361-s001.pdf]

Pasireotide is more effective than octreotide, alone or combined with everolimus on human meningioma *in vitro*

SUPPLEMENTARY MATERIALS

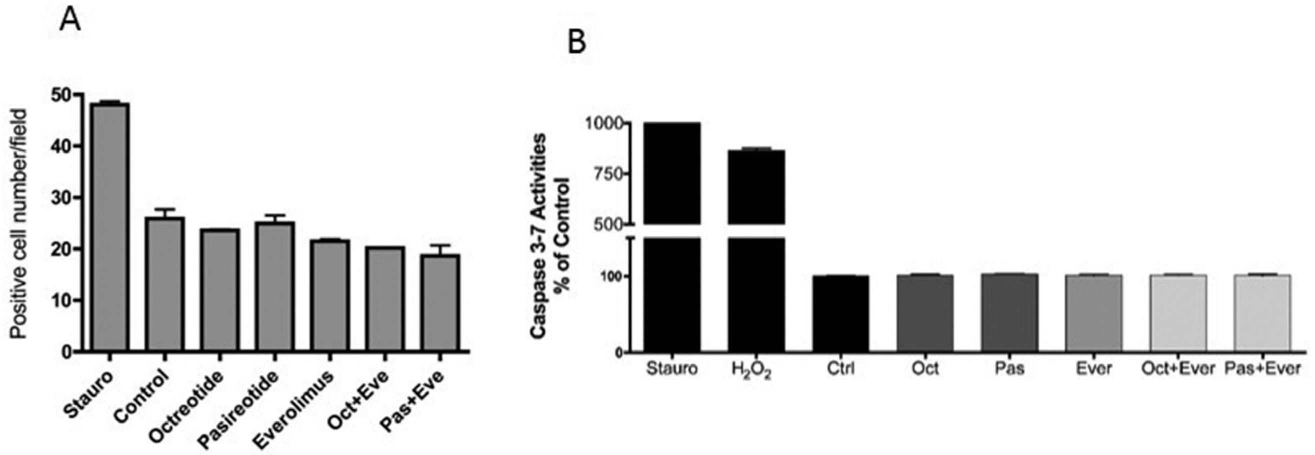

Supplementary Figure 1: Analysis of apoptosis by the TUNEL assay. (**A**) or by the measurement of caspase-3 and -7 activities (**B**) with 10−9 M octreotide (oct), pasireotide (pas), or everolimus (ever) treatments, alone or in combination. (A) TUNEL analysis of five meningioma samples (two WHO grade I and three WHO grade II) after 48 h of treatment. The number of apoptotic events was not different between treated cells and controls (nontreated cells), whereas an increase was observed in positive controls with 10−10 M staurosporine treatment (Stauro). (B) Caspase activities were measured by luminescence Caspase Glo assay in four WHO grade II meningioma samples after 24 h of treatment. No difference was observed between treated cells and control, whereas an increase was observed with 10−10 M staurosporine (Stauro) or 50 mM H_2O_2 treatment.

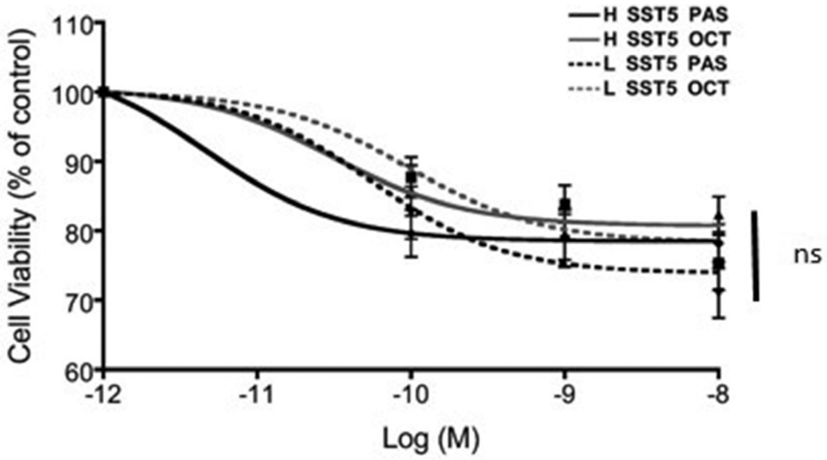

Supplementary Figure 2: Nineteen meningioma samples were divided into two groups depending on the SST5 mRNA expression level [group LSST5 (*n* = 11) with low SST5 mRNA expression level (< 0.1 copy/β-Gus) and group HSST5 (*n* = 8) with high SST5 mRNA expression level (≥ 0.1 copy/β-Gus)]. Dose–effect curves of the LSST5 group vs. the HSST5 group in the presence of octreotide (OCT) vs. pasireotide (PAS) from 10−10 to 10−8 M on cell viability estimated by Cell Titer Glo. Results are expressed as mean ± SEM percentage of cell viability vs. controls. ns: not significant.

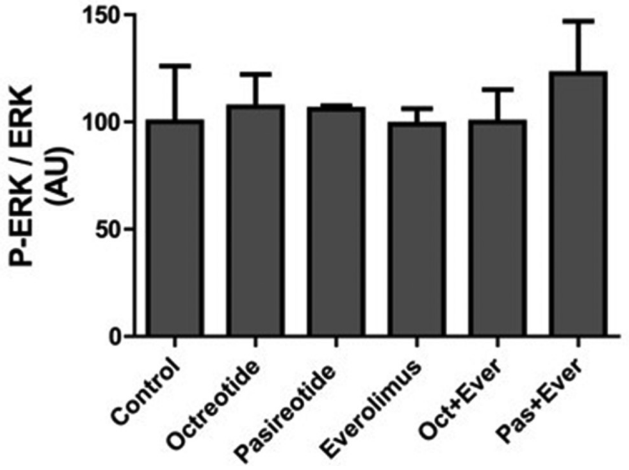

Supplementary Figure 3: Quantification from the immunoblots of pERK/ERK signals from eight meningioma samples (six WHO grade I and two WHO grade II) without treatment (control) or after 3 h of 10^{-9} octreotide, 10^{-9} pasireotide, or 10^{-9} everolimus treatment, alone or in combination. The results are represented as the mean percentage of controls (nontreated cells).

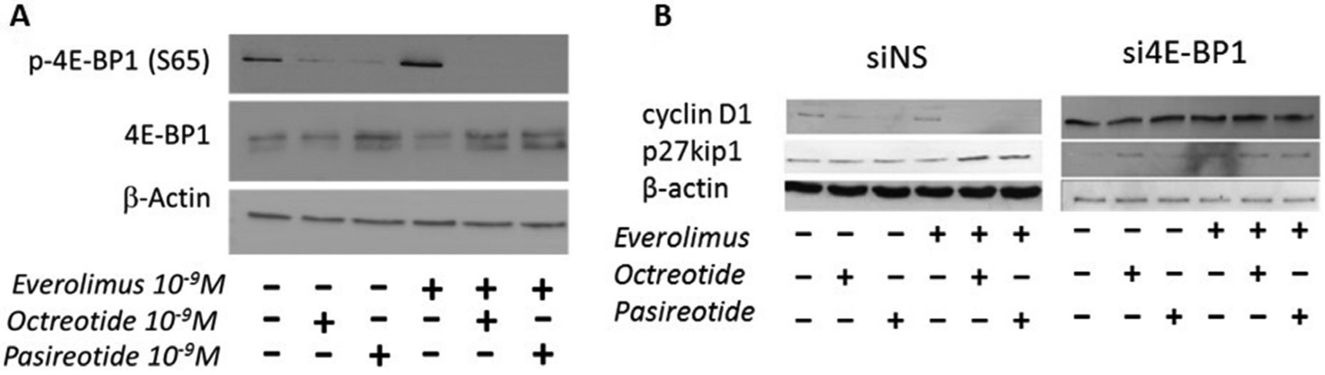

Supplementary Figure 4: Western blotting illustrating Figure 5A and Figure 5D in one meningioma sample (M57 and M59, respectively) with octreotide, pasireotide, or everolimus treatment, alone or in combination. (**A**) 4E-BP1 and the phosphorylated form p-4E-BP1 (S65); (**B**) cyclin D1 and p27kip1 in comparison to β-actin in the basal condition (siNS) or 48 h after 4E-BP siRNA (si4E-BP) transfection.

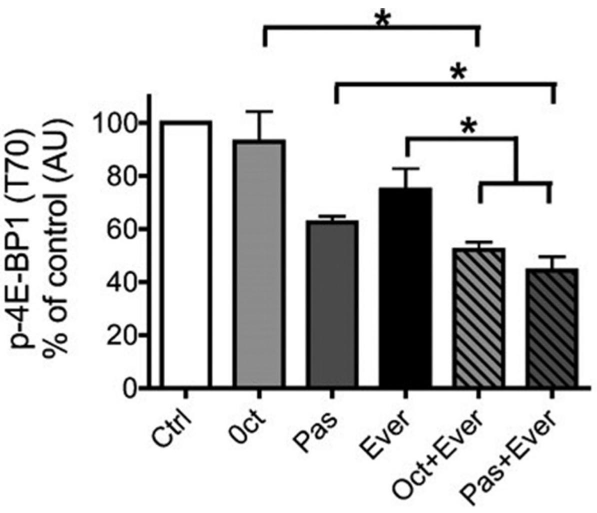

Supplementary Figure 5: Western blot analysis of 4E-BP1 phosphorylated on Thr70 [p-4E-BP1(T70)] after overnight incubation with 10−9 M pasireotide (Pas), octreotide (Oct), or everolimus (Ever), or combined treatment. The expression level of p-4E-BP1 (T70) was reported to that of total 4E-BP1. The results are represented as the mean percentage of controls (nontreated cells, ctrl) obtained from three tumor samples (two WHO grade I and one WHO grade III); *$p < 0.05$.

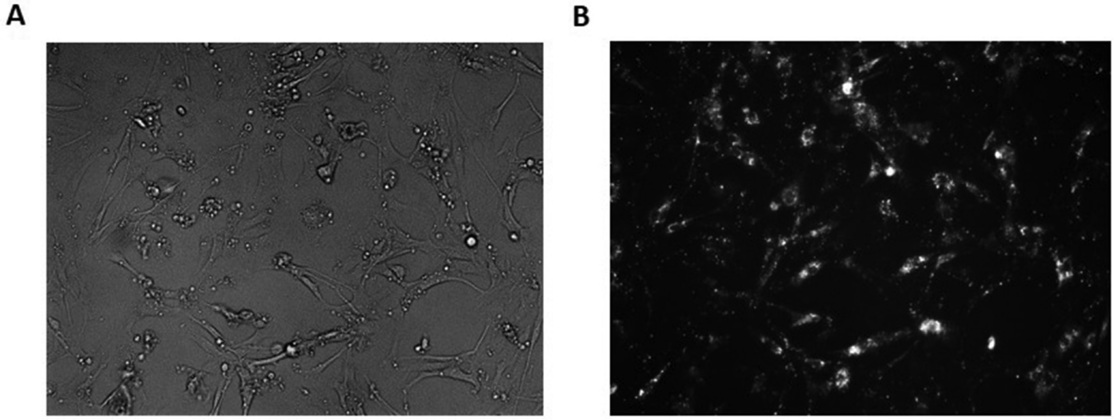

Supplementary Figure 6: The percentage of transfected cells was followed by the number of fluorescence cells using fluorescent silencer Cy-3 negative control siRNA. Meningioma cells from M59 visualized under phase contrast (**A**) or fluorescence (**B**) microscopy 48 h after transfection with 10 nM of this negative control siRNA.

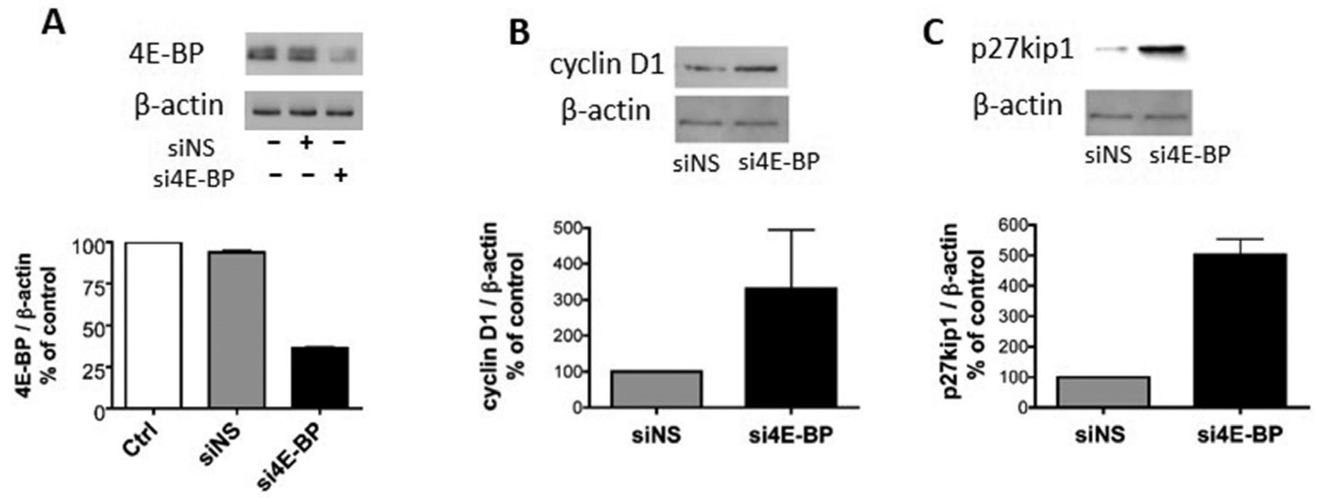

Supplementary Figure 7: Validation of 4E-BP1 siRNAs. Four human meningioma samples (three WHO grade I and one WHO grade II) were transfected with a nonspecific (siNS) or 4E-BP1 (si4E-BP) siRNA at 10 nM or were not transfected (basal condition, ctrl). 4E-BP1, cyclin D1, and p27kip1 expression levels were quantified by western blotting and were reported to the β-actin expression level. A decrease in the 4E-BP1 expression level (**A**), an increase in the cyclin D1 expression level (**B**), and an increase in the p27kip1 expression level (**C**) were observed 48 h after si4E-BP transfection in comparison with siNS. The representative immunoblot concerns M62 (A, B, C); $*p < 0.05$.

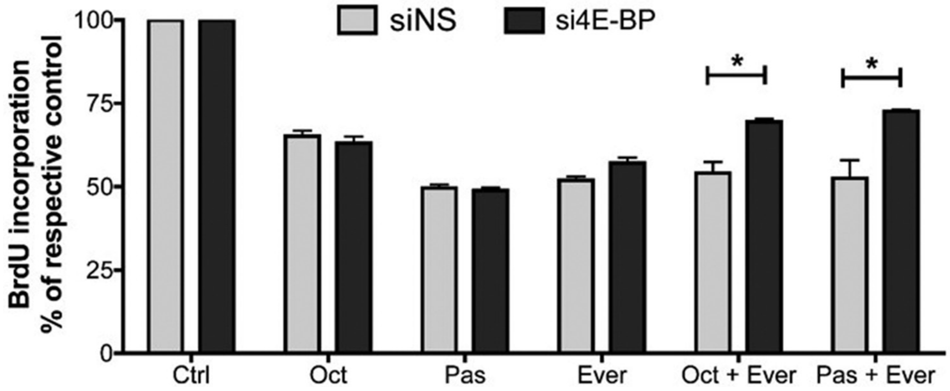

Supplementary Figure 8: Effect of 4E-BP1 siRNAs (si4E-BP) or control nonspecific siRNAs (siNS) on cell proliferation without treatment (ctrl) or with 10−9 M octreotide (Oct), 10−9 M pasireotide (Pas), or 10−9 M everolimus (Ever) treatment, alone or in combination. Cell proliferation was measured using BrdU incorporation 48 h after siRNA transfection. The results are represented as the mean percentage of the basal condition without treatment; $*p < 0.05$.

Supplementary Table 1: Characteristics of meningiomas; SST1, SST2, SST3, and SST5 mRNA expression levels; cell viability (using Cell Titer Glo assay); and cell proliferation (using BrdU incorporation) with octreotide and pasireotide treatment, both alone and in combination with everolimus

Tumor	Sub type	WHO Grade	Ki	SST2	SST1	SST3	SST5	Oct 10-9M	Pas 10-9M	Oct+Ever 10-9M	Pas+Ever 10-9M
**Cell Viability**											
M1	Anaplastic	III	20%	0,302	0,004	0.00	0.012	3	15		
M2	Transitional	I	3%	1,594	0.312	0.184	0.793	20	35		
M3	Fibrous	I	5%	8,097	0.025	0.00	0.003	22	24		
M4	Fibrous	I	3%	1,224	0.018	0.00	0.01	8	20		
M5	Psammous	I	4%	13,67	0.209	0.00	0.566	3	18		
M6	Fibrous	II	7%	0,208	0.280	0.236	0.506	30	23		
M7	Meningothelial	I	3%	4,48	0.037	0.00	0.059	36	42		
M8	Atypical	II	15%	10,84	0.002	0.00	0.007	22	31		
M9	Fibrous	I	10%	0,25	0.018			15	25.5		
M10	Fibrous	I	4%	1,233	0.03			21	32		
M11	Meningothelial	I	3%	3,836	0.327		0.735	16.5	16		
M12	Fibrous	I	5%	0,157	0.072			25	28		
M13	Psammous	I	1%	7,022	0.021	0.025	0.148	12	13		
M14	Anaplastic	III	40%	0,409	0.003	0.2	0.004	21,5	22		
M15	Atypical	II	20%	1,6	0.017	0.013	0.379	21	19		
M16	Atypical	II	10%	7,54	0.164	0.007	0.032	14,5	17		
M17	Meningothelial	I	3%	9,09	0.186	0.002	0.004	31,5	23		
M18	Meningothelial	I	7%	3,64	0.573	0,001	0,002	11,5	35		
M19	Meningothelial	I	5%	0,416	0.001	0,00	0,032	20	24.5		
M20	Meningothelial	I	3%	5,94				13	19		
M21	Fibrous	I	7%	0,48	1.425		2.562	12	14		
M22	Atypical	I	2%	0,835	0.679		0,995	2	9		
M23	Atypical	II	15%	0,719	0.158		0,044	9	43		
M24	Atypical	II	25%	1,996	1		0,967	22	6		
M25	Atypical	II	10%	4,567	0.002	0.00	0,009	6.5	27.5		
M26	Transitional	I	5%	0,138	0.003	0.00	0.003	33	29		
M27	Meningothelial	I	1%	11,05	0.152	0.00	0.006	22	27		
M28	Atypical	II	7%					16	29	33	35
M29	Meningothelial	I	4%					24	29	35	40
M30	Atypical	II	20%					22	27	37	40
M31	Meningothelial	I	5%					21	24	40	55
M32	Meningothelial	I	5%	0,118	0.008	0.001	0.002	25	24	41	55
M33	Meningothelial	I	5%	0,104	0.001	0.00	0.001	35	41	34	54
M34	Atypical	II	8%	0,244	0.001	0.00	0.002	22	23	33	35
**Cell Proliferation**											
M35	Atypical	II	7%					31	39	51	56
M36	Atypical	II	7%					40	45	56	61
M37	Transitional	I	15%					6	18	38	47
M38	Transitional	I	1%	1,276	0.013	0.00	0.00	20	32	46	52
M39	Transitional	I	2%	1,68	0.003	0.00	0.001	27	37	46	61
M40	Anaplastic	III	20%	0,758	0.001	0.00	0.009	16	24	28	41
M41	Atypical	II	8%					31	39	51	56
M42	Fibrous	I	2%					40	44	56	61
M43	Fibrous	I	7%					17	38		
M44	Atypical	II	15%					17	24		
M45	Fibrous	I	6%					22	38		

Supplementary Table 2: Principal characteristics of meningiomas tested in western blotting, siRNA studies, caspase activity measurements, and TUNEL assay

Tumor	Sub type	WHO Grade	Ki
Western Blot study			
M 37	Transitional	I	15%
M 46	Transitional	II	7%
M 47	Secretory	I	3%
M 48	Meningothelial+psammomatous	I	6%
M 49	Atypical	II	8%
M 50	Meningothelial+transitional	I	7%
M 51	Fibrous transtional	I	7%
M 52	secretory	I	0%
M 53	Meningothelial	I	3%
M 54	Meningothelial transitional atypical	II	7%
M 55	Atypical	II	25%
M 56	Atypical	II	12%
Western Blot 4E-BP1 study			
M 51	Fibrous transitional	I	7%
M 57	Anaplastic	III	40%
M 58	Fibrous	I	6%
siRNA 4E-BP1 study			
M 59	Meningothelial	I	5%
M 60	Atypical	II	12%
M 61	Fibrous	I	6%
M 62	Meningothelial	I	3%
Proliferation study			
M 63	Atypical	II	15%
M 64	Meningothelial atypical	II	3%
Caspase Glo			
M 65	Atypical	II	15%
M 66	Meningothelial Atypical	II	8%
M 67	Atypical	II	10%
M 68	Fibrous Atypical	II	8%
Tunel			
M 29	Meningothelial	I	5%
M 34	Atypical	II	8%
M 19	Meningothelial	I	5%
M 23	Atypical	II	15%
M 25	Atypical	II	10%
